# Examination of macroscopic and microscopic lesions in IBDV-infected organs and molecular characterization of IBDV VP1 gene fragments obtained from commercial broiler farms in Indonesia

**DOI:** 10.14202/vetworld.2023.1061-1070

**Published:** 2023-05-17

**Authors:** Bernike Anggun Damairia, Khrisdiana Putri, Michael Haryadi Wibowo

**Affiliations:** 1 Veterinary Science Post-Graduate Programme, Faculty of Veterinary Medicine, Universitas Gadjah Mada, Jl. Fauna 2, Karangmalang, Yogyakarta, 55281, Indonesia; 2 Widodo Makmur Unggas, Jl. Raya Cilangkap No. 58, Cilangkap, Cipayung, Jakarta 13870, Indonesia; 3 Department of Veterinary Public Health, Faculty of Veterinary Medicine, Universitas Gadjah Mada, Jl. Fauna 2, Karangmalang, Yogyakarta, 55281, Indonesia; 4 Department of Microbiology, Faculty of Veterinary Medicine, Universitas Gadjah Mada, Jl. Fauna 2, Karangmalang, Yogyakarta, 55281, Indonesia

**Keywords:** amplification, genotype, infectious bursal disease, pathological lesions

## Abstract

**Background and Aim:**

Infectious bursal disease (IBD) is an infectious immunosuppressive disease that affects young chickens. Instead of strict biosecurity practices, vaccination is used to control IBD. However, the disease has not been effectively managed. Variations in the observed clinical symptoms lead to confounding diagnoses. The study aimed to obtain pathological lesion data from chickens suspected of IBD virus (IBDV) infection by gross pathology, confirm IBDV infection through molecular diagnostics, and genotype the VP1 gene fragments of circulating IBDV in the field.

**Materials and Methods:**

The bursa of Fabricius, thymus, spleen, proventricular–ventricular junction, thigh muscles, and kidneys samples were collected from chickens suspected of IBDV infection from four commercial broiler farms in Central Java and The Yogyakarta Special Region Province between 2021 and 2022. The collected samples were examined histopathologically. Infectious bursal disease virus RNA was extracted from the bursa of Fabricius and VP1 gene was identified by reverse-transcriptase polimerase chain reaction (RT-PCR). The RT-PCR positive sample were sequenced and analyzed in Mega X for homology search and phylogenetic tree analysis.

**Results:**

Macroscopic pathological lesions in the bursa of Fabricius were demonstrated by enlarged edema and thickened plica, presence of gelatinous exudate, hemorrhage, atrophy, and caseous exudate in the lumen. Moreover, the thymus had atrophy and small gray foci were observed in the spleen. Petechiae or hemorrhage was detected on the thigh muscle, and the kidney was dull and pale. Hemorrhage in the proventricular–ventricular junction was distinct. The histopathological examination of the bursa of Fabricius showed follicular vacuolization, edema, heterophilic infiltration, follicular atrophy, congestion, and hemorrhage. The thymus and spleen showed the presence of multifocal necrosis. Hemorrhage was observed in thigh muscle and mucosal part of proventricular–ventricular junction. Vacuolization was seen in renal tubules (nephrosis). Reverse transcriptase-PCR of 26 bursa of Fabricius samples from chickens suspected of IBDV infection showed four negative and 22 positive samples. Phylogenetic analysis of the VP1 gene fragment has indicated very virulent IBD (vvIBD) and belonged to B2 genotype.

**Conclusion:**

Infectious bursal diseases virus infection in broiler chicken generated macroscopic and microscopic primary lesions in the bursa of Fabricius and thigh muscle. Other organs such as the spleen, thymus, proventricular–ventricular junction, and kidney, were also involved. Molecular analysis of the VP1 gene confirmed the causative agent and grouped the virus into vvIBD and B2 genotype. All samples were collected from vaccinated birds therefore, the efficacy of available vaccine is required for urgent evaluation. Since most studies only focused on VP1, further exploration on VP2 gene is suggested notably for new-generation vaccines. Monitoring clinical signs’ transformation over time could assist field diagnostics.

## Introduction

Infectious bursal disease (IBD) or Gumboro, is an immunosuppressive disease that affects young chickens. The virus is an RNA virus of the genus Avibirnavirus of the *Birnaviridae* family. The virus has an icosahedral symmetrical shape, is 55–65 nm in diameter, and is non-clustered. The viral genome is dsRNA with two segments: A and B. Segment A (3,261 bp) encodes VP5 and a precursor polyprotein (VP2-VP3-VP4). This protein is autocatalytically cleaved by the viral protease VP4 into the main structural proteins of the virion VP2 and VP3. Segment A also encodes a non-structural protein VP5. [1, 2]. VP2 also plays a crucial role in virulence, cell tropism, pathogenicity phenotype, and protective immunity [3]. Segment B (2,827 bp) encodes VP1, an enzyme with RNA-dependent-RNA polymerase activity [1], which is crucial for the replication and genetic evolution of IBD virus (IBDV) [4]. Immature B-lymphocytes in the follicles of the bursa of Fabricius are targets of IBDV since they contain immunoglobulin M specific for viral infection [5, 6]. Actively proliferating cells are the primary target of IBDV. The affinity of IBDV is greater toward immature B-lymphocytes [7]. In B-lymphocytes, Gumboro virus inhibits proliferation and promotes apoptosis [8]. Allan *et al*. [9] reported that defects in B-lymphocytes lead to impaired response to vaccination. Chickens infected with Gumboro virus at an early stage may suffer from reduced antibody response, increasing susceptibility to other diseases. The clinical manifestations of and mortality caused by IBD vary depending on virulence, infection dose, age, chicken type, and presence of passive immunity [10]. The very virulent IBD (vvIBD) type causes severe lesions to immune-related organs, such as the thymus, spleen, bursa of Fabricius, as well as the liver, kidneys, heart, proventriculus, gizzard, and ceca tonsil [11]. Lesions in the pectoral and thigh muscles, including dark and dehydrated muscles, and hemorrhage have been observed [1]. A very virulent infectious bursal disease virus (vvIBDV) can cause extensive thymus cell necrosis. Further reports of IBD cases exhibited edema, lymphoid cell follicle draining, also follicle connective tissue fibrosis, and proliferation of endothelial reticular cells [12, 13]. Chicken mortality due to IBDV infection was 59.09% in broilers and 25.08% in the commercial layer. In vvIBDV, mortality rate may reach 35%–75% [14].

Infectious bursal disease virus is classified into pathogenic serotype I and non-pathogenic serotype II. Based on pathogenicity, IBDV belonging to serotype I is classified into mild, intermediate, intermediate plus, classical, variant, and very virulent strains [15]. Due to the increasingly complex development of IBDV, classification based on antigenicity and pathogenicity has become more complicated. Classification based on gene sequence data is more consistent and reliable [16]. To add, current report on IBD genotyping mostly focus on VP2 only [17]. Seven genogroups have been reported thus far. A1 is a classic IBDV, A2 is an antigenic variant virus, A3 is a vvIBDV, and A4 is a distinct (d) IBDV, characterized by 222S, 272T, 289P, 290I, and 296F. A5 is a variant virus or a classic recombinant IBDV, A6 is an Italian variant, and A7 is an Australian variant [16, 18]. The identified IBDV strain enters the A8 genogroup [19], and IBDV serotype 2 is classified in the all genotype [19]. Initially, the evolutionary analysis of IBDV focused on the role of VP2 in virulence, antigenicity, and tropism. The function of segment B, specifically VP1, attract great attention. Several studies have shown the role of VP1 in the evolution and emergence of vvIBDV [4]. Therefore, a molecular epidemiological investigation should include VP1 in genogroup analysis [19]. A previous study [19] has proposed genotyping based on segment B of VP1 gene. Serotype I is grouped into cluster B1 (including non-very virulent strain), B2 (vvIBDV), B3 (IBDV originated in other poultry or wild-bird reservoirs), and B4 (European lineage IBDV). Serotype II is classified into the BII genogroup [19].

In Indonesia, subclinical IBD cases in 1991 caused low mortality. The first outbreak of IBD reported an acute increase and expansion of IBD cases, with high mortality reaching 25% in broilers and 60% in laying hens [14]. Based on VP2 gene, IBDV Indonesia isolates are majority grouped in vvIBDV, although classical viruses were also detected [20, 21]. Infectious bursal disease outbreaks have been reported in many areas of Indonesia, although vaccination and stringent biosecurity have long been used in farms. Diagnosis in the field is arduous to perform because classical signs are not perpetually present. In addition, genetic characterization of latest IBDV Indonesia VP1 gene is lacking. This gene is important in virus encapsidation, virulence, and replication. Genetic data would assist control strategies development and support field diagnostics.

Thus, this study aimed to obtain pathological lesion data and VP1 gene characterization of the latest cases of IBD in commercial broiler farms in Indonesia. This is the first genotype analysis of IBDV VP1 gene from Indonesia.

## Materials and Methods

### Ethical approval

The samples were obtained from IBDV case in commercial broiler farms. There were no challenge or live animal experiment involved. Thus, ethical clearance is not required. The method has been reviewed and obtained approval from Faculty of Veterinary Medicine, Universitas Gadjah Mada, Indonesia (approval number 4100/UN1/FKH/TU/2022).

### Study period and location

The study was conducted from December 2021 to October 2022. The samples were collected from commercial broiler farms in the Special Region of Yogyakarta (DIY) and Central Java. The histopathology slides were processed at the Wates Disease Investigation Center, Yogyakarta. The RT-PCR test was carried out at the Department of Microbiology, Faculty of Veterinary Medicine, Universitas Gadjah Mada University.

### Samples

Samples were obtained from chickens suspected of IBDV by gross pathology in four commercial broiler farms: Sragen, Wonogiri, Batang District of Central Java Province, and Sleman District of Yogyakarta Special Region Province. A total of five chickens from each farm with indicative clinical symptoms of IBD were selected. The clinical signs were recorded, and necropsies were performed to observe lesions on the bursa of Fabricius, thymus, spleen, proventricular–ventricular junction, thigh muscles, and kidneys. The collected organs were preserved in 10% formalin prior to histology slides processing. A part of the bursa of Fabricius was stored at −20°C for molecular analysis.

Histology slides were processed at the Wates Disease Investigation Centre, Yogyakarta Special Region Province, according to the standard protocols of the institution. Histopathology examination was conducted at the Department of Pathology, Faculty of Veterinary Medicine, UGM.

### RNA extraction, reverse transcriptase polymerase chain reaction (RT-PCR), and sequencing

All samples were tested for the VP1 gene by RT-PCR. The genetic material of IBDV was extracted from the bursa of Fabricius using Geneaid Viral Nucleic Acid Extraction Kit II (Geneaid, Taiwan) according to the manufacturer. The VP1 gene was amplified using specific primers: Forward 5’-cta cgg gag tgg gac cta ca-3’ and reverse 5’-acc acg tgt tgg agt gaa ca-3’, which yielded a 749-bp amplicon [22]. The polymerase chain reaction was performed in 50-μL reaction volume, using MyTaq One-Step RT-PCR Kit (Bioline Reagents Ltd., United Kingdom). Reverse transcriptase-PCR was performed under the following cycling conditions: transcription at 50°C for 30 min; pre-denaturation at 95°C for 5 min; 40 cycles of denaturation at 94°C for 45 s, annealing at 62°C for 45 s, extension at 72°C for 45 s; and final extension at 72°C for 5 min. The PCR product was visualized by electrophoresis on 1.2% agarose in 1× Tris-borate-EDTA buffer. Amplified samples were sequenced at First BASE (Apical Scientific, Selangor, Malaysia) and analyzed using Mega X (https://www.megasoftware.net/) [23]. The sequences reading were compared with VP1 gene sequences of IBDV available in GenBank [24].

## Results

### Lesion examination

Organ samples were collected from 26 chickens with signs of depression, trembling, whitish diarrhea, feather loss, paralysis, and dirty cloaca, with mortality rates between 3.82%–20.41%. The organs evaluated included the bursa of Fabricius, thymus, spleen, proventricular–ventricular junction, thigh muscles, and kidneys. All observed changes were recorded and are summarized in Table-1. Changes were seen in the bursa of Fabricius (100%), spleen (92.31%), thymus (7.69%), proventricular–ventricular junction (42.31%), thigh muscles (100%), and kidneys (57.69%).

**Table-1:** Total involved organs showing lesion in IBD cases from commercial broiler farm (n = 26).

Organs	Total	Percentage
Bursa of Fabricious	26	100.00
Spleen	24	92.31
Thymus	2	7.69
Proventricular-ventricular junction	11	42.31
Thigh muscle	26	100.00
Kidneys	15	57.69

**Figure-1:**
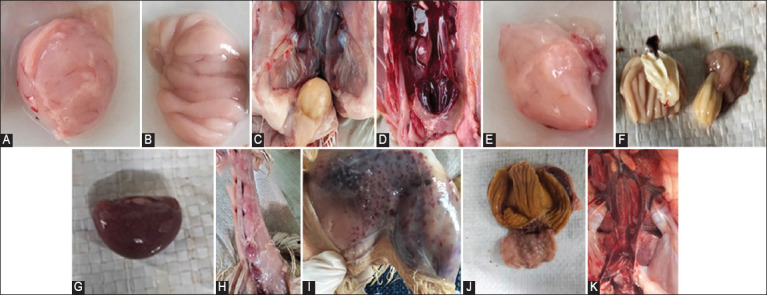
Gross pathology of IBDV-infected organs. (A) Enlarged and edematous bursa of Fabricius, (B) Thickened plica bursa of Fabricius, (C) Gelatinous serosal surface of bursa of Fabricius (acute), (D) Hemorrhage in the bursa (acute), (E) Atrophy bursa of Fabricius (chronic), (F) Atrophy and cheese mass in the lumen bursa of Fabricius (chronic), (G) White-gray spots on the spleen, (H) Atrophy and thymus hemorrhage, (I) Hemorrhage in the thigh muscle, (J) Hemorrhage at proventricular-ventricular junction, and (K) Swelling and pallor of the kidneys.

Macroscopic lesions of involved organs in IBD cases were collected from commercial broiler farms are shown in Table-2. The bursa of Fabricius was enlarged, with edema and thickened plica (3.85%) (Figures-1A and B), gelatinous (19.23%) (Figure-1C), with hemorrhage (15.38%) (Figure-1D), atrophy (57.69%) (Figure-1E), and accumulation of a cheesy mass in the lumen (3.85%) (Figure-1F). In other organs showing lesions, we observed gray-white spots on the spleen (92.31%) (Figure-1G), atrophy of the thymus (7.69%) (Figure-1H), thigh muscle hemorrhage (46.15%) (Figure-1I), proventricular–ventricular junction petechiae (53.85%) (Figure-1J), and pale and swollen kidneys (57.69%) (Figure-1K).

**Table-2:** Macroscopic lesions of involved organs in IBD cases collected from commercial broiler farms (n = 26).

Organs	Lesion	Total	Percentage
Bursa of Fabricius	Enlarged, edema, and thickened plica	1	3.85
	Gelatinous mass	5	3.85
	Hemorrhage	4	15.38
	Atrophy	13	50.00
	Necrotizing caseosa in the lumen	3	11.54
Spleen	Grey-white spot	24	92.31
Thymus	Atrophy	2	7.69
Proventricular-ventricular junction	Hemorrhage	11	42.31
Thigh muscle	Petechiae	14	53.85
	Hemorrhage	12	46.15
Kidneys	Pale and swollen	15	57.69

### Histopathological analysis

**Figure-2:**
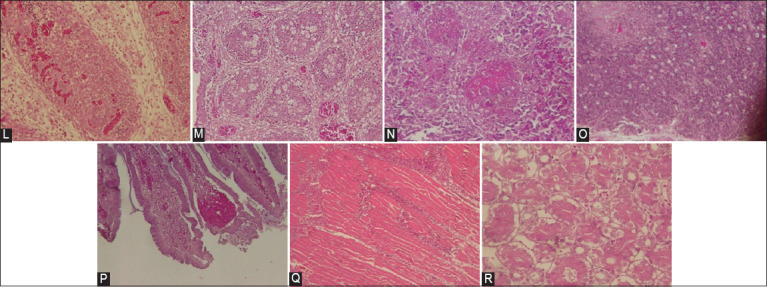
Histopathology reading of IBDV-infected bursa of Fabricius. (L) Acute form: edema, hemorrhage, congestion, and heterophile infiltration, (M) Chronic form: follicular atrophy, lymphoid necrosis, vacuolization of the follicular bursa, proliferation of fibroblasts and connective tissue in interfollicular tissue, (N) Multifocal follicle necrosis occurs between the white pulp in the spleen, (O) Multifocal follicle necrosis occurs between a sinus in the thymus. (P) Hemorrhage of the pars mucosal proventricular-ventricular junction and, (Q) Hemorrhage occurs between the thigh muscle fibers, and (R) Renal vacuolization (nephrosis). 200× magnification, hematoxylin eosin staining.

Obtained histopathological changes involving organs are grouped according to lesion type (Table-3). Observed histological changes in the bursa of Fabricius (Figure-2) included follicular vacuolization (23.08%), edema (3.85%), heterophile infiltration (3.85%), follicular atrophy (61.54%), congestion (15.38%), and hemorrhage (15.38%). Pathological changes were also observed in other organs including necrosis with lymphocyte accumulation in the white pulp of the spleen (57.69%), multifocal follicle necrosis of the thymus (7.69%), proventricular–ventricular junction pars mucosal hemorrhage (38.46%), petechiae (53.85%), hemorrhage (46.15%) of the thigh muscle, and vacuolization in the renal tubules (53.85%).

**Table-3:** The percentage of lesion type in organs affected by IBD (n = 26).

Organ	Detailed lesion	Total	Percentage
Bursa fabricius	Follicular vacuolization	6	23.08
	Edema	1	3.85
	Heterophile infiltration	1	3.85
	Follicular atrophy	16	61.54
	Congestion	4	15.38
	Hemorrhage	4	15.38
Spleen	Multifocal follicle necrosis in the white pulp	15	57.69
Thymus	Multifocal follicle necrosis	2	7.69
Proventricular-ventricular junction	Hemorrhage of the mucosal pars	10	38.46
Thigh muscle	Petechiae	14	53.85
	Hemorrhage	12	46.15
Kidneys	Vacuolization of renal tubules (nephrosis)	14	53.85

### Sequence and phylogram analysis

**Figure-3:**
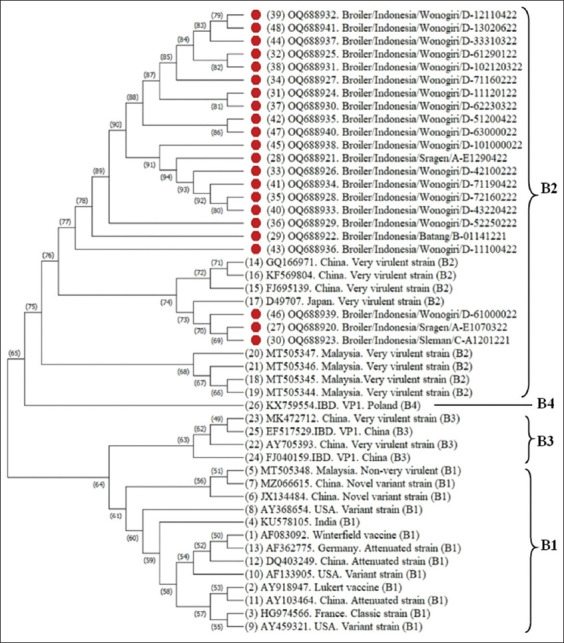
Phylogenetic tree analysis of the VP1 gene fragment of infectious bursal disease virus with bootstrap value of 1000×. Red dot represents the virus under study.

The RT-PCR analysis revealed 22 out of 26 bursal samples positive for the VP1 gene. The sequences were analyzed against the VP1 gene sequence available in GenBank. Homology analysis indicated variations in amino acid residues 166, 167, 360, 399, 401–404, 410, and 412 (Table-4). The phylogram (Figure-3) formed two subclusters within the B2 genotype. Furthermore, 19 out of 22 samples fell into a separate cluster from vvIBD in the B2 genotype, whereas three samples clustered with the vvIBD reference virus genogroup B2 from China, and Japan.

**Table-4:** Amino acid variation of VP1 gene 166–412 of IBD virus in the study compared to VP1 gene sequence available in GenBank.

No.	Accession No./Strain/Genotype	Amino acid substitution at position VP1 gene													

		166	167	242	287	360	390	393	399	401	402	403	404	410	412
1	AF083092/Winterfield vaccine/B1	S	G	D	T	P	L	E	V	A	D	N	I	N	W
2	AY918947/Lukert vaccine/B1														
3	HG974566/France. Classic strain/B1														
4	KU578105/India/B1						M	D							
5	MT505348/Malaysia. Non-very virulent/B1														
6	JX134484/China. Novel variant strain/B1														
7	MZ066615/China. Novel variant strain/B1														
8	AY368654/USA. Variant strain/B1														
9	AY459321/USA. Variant strain/B1														
10	AF133905/USA. Variant strain/B1														
11	AY103464/China. Attenuated strain/B1														
12	DQ403249/China. Attenuated strain/B1														
13	AF362775/Germany. Attenuated strain/B1														
14	GQ166971/China. Very virulent strain/B2			E	A	.	M	D							
15	FJ695139/China. Very virulent strain/B2			E	A	S	M	D							
16	KF569804/China. Very virulent strain/B2			E	A	.	M	D							
17	D49707/Japan. Very virulent strain/B2			E	A	.	M	V							
18	MT505345/Malaysia. Very virulent strain/B2			E	A		M	D							
19	MT505344/Malaysia. Very virulent strain/B2			E	A		M	D							
20	MT505347/Malaysia. Very virulent strain/B2			E	A		M	D							
21	MT505346/Malaysia. Very virulent strain/B2			E	A		M	D							
22	AY705393/China. Very virulent strain/B3		.	.	A										
23	MK472712/China. Very virulent strain/B3				A										
24	FJ040159/China. Very virulent strain/B3				A										
25	EF517529/China. Very virulent strain/B3				A										
26	KX759554/Poland. Very virulent strain/B4				A	.	M								
27	OQ688920/Broiler/Indonesia/Sragen/A-E1070322	R	W	E	A	S	M	D							
28	OQ688921/Broiler/Indonesia/Sragen/A-E1290422	.	.	E	A	.	M	D							
29	OQ688922/Broiler/Indonesia/Batang/B-01141221	.	.	E	A	.	M	D							
30	OQ688923/Broiler/Indonesia/Sleman/C-A1201221	.	.	E	A	S	M	D							
31	OQ688924/Broiler/Indonesia/Wonogiri/D-11120122	R	W	E	A	.	M	D						T	.
32	OQ688925/Broiler/Indonesia/Wonogiri/D-61290122	E	.	E	A	.	M	D						T	.
33	OQ688926/Broiler/Indonesia/Wonogiri/D-42100222	.	.	E	A	.	M	D	L						
34	OQ688927/Broiler/Indonesia/Wonogiri/D-71160222	E		E	A		M	D	.						
35	OQ688928/Broiler/Indonesia/Wonogiri/D-72160222			E	A		M	D				Y	L		
36	OQ688929/Broiler/Indonesia/Wonogiri/D-52250222			E	A		M	D							
37	OQ688930/Broiler/Indonesia/Wonogiri/D-62230322	R	W	E	A		M	D				.	.	T	.
38	OQ688931/Broiler/Indonesia/Wonogiri/D-102120322	K	.	E	A		M	D				.	.	T	.
39	OQ688932/Broiler/Indonesia/Wonogiri/D-12110422	E	.	E	A		M	D	.	.	.	.	.	T	.
40	OQ688933/Broiler/Indonesia/Wonogiri/D-43220422	.	.	E	A		M	D	.	V	H	I	L	.	.
41	OQ688934/Broiler/Indonesia/Wonogiri/D-71190422			E	A		M	D	.	.	.	.	.	.	.
42	OQ688935/Broiler/Indonesia/Wonogiri/D-51200422			E	A		M	D						T	
43	OQ688936/Broiler/Indonesia/Wonogiri/D-11100422			E	A	S	M	D							C
44	OQ688937/Broiler/Indonesia/Wonogiri/D-33310322	K		E	A		M	D						T	
45	OQ688938/Broiler/Indonesia/Wonogiri/D-101000022			E	A		M	D							
46	OQ688939/Broiler/Indonesia/Wonogiri/D-61000022			E	A	S	M	D						T	
47	OQ688940/Broiler/Indonesia/Wonogiri/D-63000022			E	A		M	D						T	
48	OQ688941/Broiler/Indonesia/Wonogiri/D-13020622	E		E	A		M	D						T	

## Discussion

The clinical symptoms observed in this study are consistent with those reported elsewhere. Chickens infected with IBDV exhibit general weakness, whitish diarrhea, dirty cloaca, and tremors [25–28]. These clinical symptoms are distinct from those of similar diseases, such as inclusion bodies hepatitis (IBH), reported by Wibowo *et al*. [29], or experimental study post-challenge [30]. Furthermore, chickens with coccidiosis exhibit general weakness, sudden death, and depression without thigh muscle hemorrhage and bursal edema [31]. The mortality rate in this study is between 3.82% to 20.41%. Zannah *et al*. [26] reported the mortality rate of IBDV infection was 4.65%. However, higher mortality rates have been claimed to 40% [15], 51% [25], and 77.73%–98.56% [32].

The bursa of Fabricius is the primary organ affected by IBDV infection and generally presents with inflammation, edema, hyperemia, hemorrhage, and atrophy [33]. Bursal atrophy may occur 3–5 d after infection [34]. In sub-chronic or chronic IBD, bursal weight was reported to be significantly decreased [35]. This is due to atrophy following recovery from infection. Atrophy was also detected in other lymphoid organs, such as the thymus [36]. Bursal atrophy was also found in Marek’s disease and chicken anemia virus infection [31]. In addition to the major immune organ bursa of Fabricius, other organs, such as the thymus, spleen, proventricular–ventricular junction, kidney, and thigh muscles, are targets of IBDV infection [37]. Akter *et al*. [38] consistently reported hemorrhage in the proventricular–ventricular junction and thigh muscle, as well as swollen kidneys. Wibowo *et al*. [29] reported swelling and hemorrhaging of the bursa of Fabricius, swollen kidneys with uric acid deposits, and hemorrhaging in IBH. A pale and swollen liver with multifocal necrosis is always present in IBH. Hydro pericarditis is frequently seen in cases of IBH [29]. Liver lesions are distinct characteristic that differentiates between IBH and IBD [29]. In an experimental mix infection model of IBD and IBH, liver necrosis and bursa of Fabricius atrophy were seen after 5 d post infection [39].

Histopathological lesions from bursal samples (Table-2) exhibited follicular vacuolization (23.08%), edema (3.85%), heterophile infiltration (3.85%), follicle atrophy (61.54%), congestion (15.38%), and hemorrhage (15.38%). Bursal hemorrhage was less observed than atrophy, which may be related to sample collection time at the post-peak period of the outbreak [40–42]. Lymphocyte defects and vacuolization of the bursa of Fabricius in this study are consistent with Kulsum *et al* [43]. Furthermore, histopathological studies have revealed severe lymphoid depletion and heterophile and macrophage infiltration in the interfollicular space of the bursa of Fabricius [38, 43]. Widespread acute lymphoid necrosis, follicular hemorrhage, and stromal edema in the bursa of Fabricius were identified as an acute form of IBD [44]. Challenge experimental study of IBDV field isolate has been reported to generate a liver lesion. The liver of 25-day-old chickens inoculated with the field isolate of IBDV exhibited congestion on day 1 post-inoculation, followed by localized necrosis, hemorrhage, and fatty degeneration [45]. However, liver lesion is uncommon in IBD field cases (interviews report with poultry practitioner). The IBD lesion could be differentiated with IBH which mostly exhibits swollen and multifocal necrosis liver [46]. Other differential diagnoses of IBD are avian coccidiosis, chicken anemia virus infection, mycotoxicosis, and Marek’s disease [31]. Multifocal follicle necrosis was observed in the thymus and spleen by Zahid *et al*. [47]. A previous study has reported renal tubule hypertrophy in the kidney on days 3–4 post-inoculation of IBD. The proximal segment of the tubules had empty areas (vacuoles) that extended to the lumen of the distal tubule [47]. The renal tubules exhibited necrosis and the glomerulus showed atrophy and an increased size of capsular (vacuolization) [48].

The molecular analysis could only confirm 22 samples by RT-PCR, possibly the viral load was too low because samples were collected after peak infection [49]. This evidence is supported by macroscopic and microscopic analyses indicated chronic infection of the bursa of Fabricius. Infectious bursal disease virus load concentrations peaked 3–4 days after infection; however, chronic lesions in the bursa of Fabricius occurred 7–10 days after infection [49].

Based on sequence analysis, some variations were detected in amino acid residues D242E, T287A, L360M, and E393D of all isolates in this study. Mutations in residues D242E, T287A, L360M, and E393D were reported to alter pathogenicity from classic to very virulent strain [50, 51, 52, 53].

The phylogram of the VP1 gene included IBDVs from serotype I (B1, B2, B3, B4) and serotype II (BII). Based on the VP1 gene sequence, IBDV is divided into genogroups B1 (non-vvIBD) and B2 (vvIBD) [54]. By contrast, Zhang *et al*. [54] divided IBD into two genotype groups: vvIBD and non-vvIBD. Phylogenetic tree analysis showed 3 out of 22 samples were included in genotype B2: Broiler/Indonesia/Wonogiri/D-61000022 (Acc. number: OQ688939), Broiler/Indonesia/Sragen/A-E1070322 (Acc. number OQ688920), and Broiler/Indonesia/Sleman/C-A1201221 (Acc. number: OQ688923) grouped in with very virulent strains from Japan (Acc. Number: D49707) and very virulent strains from China (Acc. number: GQ166971, KF569804, and FJ695139). Amino acid residues D242E, T287A, L390M, and E393D are similar between Japan and China virulent strains. The remaining 19 samples were forming a new subcluster. These isolates showed amino acid residue mutation not only at D242E, T287A, L390M, and E393D but also S166R, G167W, S166E, S166K, P360S, V399L, N403Y, I404L, N410T, A401V, D402H, N403I, I404L, W412C. According to Michel and Jackwood [18], the VP1 gene Indonesia IBD isolates (accession numbers MF142467, MF142480, MF142494, and MF142496) was classified as very virulent. This report is also supported by Mahardika and Parede [20]; Wibowo *et al*. [21] that the majority of circulating IBDV in commercial farms in Indonesia was classified as vvIBD based on the VP2 gene in genogroup 3. This has provided novel insights into circulating IBDV in Indonesia. Müller *et al*. [7] suggested reassortants or recombination of serotype 1/serotype 2 and controlled attenuated vvIBDV as a promising new-generation IBD vaccine. Since the emergence of vvIBD, disease control by vaccination, with high-level biosecurity and good management practice, has become more challenging [55].

Our study has limitations. The genetic properties of identified vvIBD strains must be further explored. Moreover, this study should include a larger area of samples and include cases from layer farms.

## Conclusion

Broiler chickens infected with IBDV showed specific macroscopic and microscopic lesions in the bursa of Fabricius and thigh muscle. Moreover, other organs such as the spleen, thymus, proventricular–ventricular junction, and kidney were affected. Molecular analysis of the VP1 gene fragment has grouped the virus into vvIBD of B2 genotype. This study has provided evidence of the latest IBDV genotype circulating in broilers in Indonesia. Most studies on IBDV have focused on the VP1 gene; however, some studies have suggested using VP2 for generating more effective IBDV vaccines. Periodic monitoring of the latest isolate for the probability of genome mutation and clinical signs update of IBD field cases. Because all samples were obtained from vaccinated chickens, our findings provide a new outlook regarding vaccine efficacy.

## Authors’ Contributions

BAD: Collected samples, performed laboratory work, analyzed the data, and drafted the manuscript. KP: Supervised the molecular work and reviewed the manuscript. MHW: Designed the study, supervised all the laboratory work and the analysis, and reviewed the manuscript. All authors have read, reviewed, and approved the final manuscript.

## References

[1] Eterradosi N, Saif Y.M (2013). Diseases of Poultry.

[2] Nagarajan M.M, Kibenge F.S (1997). Infectious bursal disease virus:A review of molecular basis for variations in antigenicity and virulence. Can. J. Vet. Res.

[3] Brandt M, Yao K, Liu M, Heckert R.A, Vakharia V.N (2001). Molecular determinants of virulence, cell tropism, and pathogenic phenotype of infectious bursal disease virus. J. Virol.

[4] Yu F, Ren X, Wang X, Qi X, Song J, Gao Y, Qin L, Gao H, Wang X (2013). A single amino acid V4I substitution in VP1 attenuates virulence of very virulent infectious bursal disease virus (vvIBDV) in SPF chickens and increases replication in CEF cells. Virology.

[5] Kaufer I, Weiss E (1980). Significance of bursa of Fabricius as a target organ in infectious bursal disease of chickens. Infect. Immun.

[6] Sharma J.M, Kim I.J, Rautenschlein S, Yeh H.Y (2000). Infectious bursal disease virus of chickens:Pathogenesis and immunosuppression. Dev. Comp. Immunol.

[7] Müller H, Islam M.R, Raue R (2003). Research on infectious bursal disease--the past, the present and the future. Vet. Microbiol.

[8] Sivanandan V, Maheswaran S.K (1980). Immune profile of infectious bursal disease (IBD). II. Effect of IBD virus on pokeweed-mitogen-stimulated peripheral blood lymphocytes of chickens. Avian Dis.

[9] Allan W.H, Faragher J.T, Cullen G.A (1972). Immunosuppression by the infectious bursal agent in chickens immunised against Newcastle disease. Vet. Rec.

[10] Ingrao F, Rauw F, Lambrecht B, Van den Berg T (2013). Infectious bursal disease:A complex host-pathogen interaction. Dev. Comp. Immunol..

[11] Oladele O.A, Adene D.F, Obi T.U, Nottidge H.O (2009). Comparative susceptibility of chickens, turkeys and ducks to infectious bursal disease virus using immunohistochemistry. Vet. Res. Commun.

[12] Nunoya T, Otaki Y, Tajima M, Hiraga M, Saito T (1992). Occurrence of acute infectious bursal disease with high mortality in Japan and pathogenicity of field isolates in specific-pathogen-free chickens. Avian Dis.

[13] Park J.H, Sung H.W, Yoon B.I, Kwon H.M (2009). Protection of chicken against very virulent IBDV provided by in ovo priming with DNA vaccine and boosting with killed vaccine and the adjuvant effects of plasmid-encoded chicken interleukin-2 and interferon-g. J. Vet. Sci..

[14] Parede L.H, Sapats S, Gould G, Rudd M, Lowther S, Ignjatovic J (2003). Characterization of infectious bursal disease virus isolates from Indonesia indicates the existence of very virulent strains with unique genetic changes. Avian Pathol..

[15] Orakpoghenor O, Oladele S.B, Abdu P.A (2020). Infectious Bursal Disease:Transmission, pathogenesis, pathology and control-an overview. Worlds Poult. Sci. J..

[16] Jackwood D.J, Schat K.A, Michel L.O, De Wit S (2018). A proposed nomenclature for infectious bursal disease virus isolates. Avian Pathol.

[17] Jackwood D.J, Sommer-Wagner S.E (2010). Detection and characterization of infectious bursal disease viruses in broilers at processing. Prev. Vet. Med..

[18] Michel L.O, Jackwood D.J (2017). Classification of infectious bursal disease virus into genogroups. Arch. Virol..

[19] Yu-Long W, Lin-Jin F, Nan J, Li G, Kai L, Yu-Long G, Chang-Jun L, Hong-Yu C, Qing P, Yan-Ping Z, Xiao-Mei W, Xiao-Le Q (2021). An improved scheme for infectious bursal disease virus genotype classification based on both genome-segments A and B. J. Integr. Agric.

[20] Mahardika I.G.N.K, Parede L (2008). Phylogenetic analysis of nucleotide sequence of hypervariable fragment of VP2 of infectious bursal disease virus isolated in Indonesia. J. Vet.

[21] Wibowo M.H, Anggoro D, Wibowo S.E, Santosa P.E, Amanu S, Asmara W (2017). Analisis fragmen gen VP2 virus infectious bursal diseases yang diisolasi dari peternakan ayam komersial (Analyses of VP2 Gene Fragment of Infectious Bursal Diseases Viruses Isolated from Commercial Poultry Farm). Acta Vet. Indones..

[22] Ashraf S, Tang Y, Saif Y.M (2007). Development of differential RT-PCR assays and molecular characterization of the complete VP1 gene of five strains of very virulent infectious bursal disease virus. Avian Dis.

[23] Kumar S, Stecher G, Li M, Knyaz C, Tamura K (2018). MEGA X:Molecular Evolutionary Genetics Analysis across computing platforms. Mol. Biol. Evol..

[24] Benson D.A, Karsch-Mizrachi I, Clark K, Lipman D.J, Ostell J, Sayers E.W (2012). GenBank. Nucleic Acids Res.

[25] Omer M.G, Khalafalla A.I (2022). Epidemiology and laboratory diagnosis of very virulent infectious bursal disease virus in vaccinated chickens in Khartoum, Sudan. Open Vet. J.

[26] Zannah M, Awaludin A, Rukmi D.L, Nusantoro S, Kusuma S.B (2020). Case study on genesis infectious bursal disease (IBD) on broiler chickens at PT. Aretha Nusantara Farm Bandung. J. Livest. Sci. Prod..

[27] Liang J, Yin Y, Qin T, Yang Q (2015). Chicken bone marrow-derived dendritic cells maturation in response to infectious bursal disease virus. Vet. Immunol. Immunopathol..

[28] Oluwayelu D.O, Emikpe B.O, Ikheloa J.O, Fagbohum O.A, Adeniran G.A (2002). The pathology of infectious bursal disease in crossbreeds of harco cocks and indigenous Nigerian hens. Afr. J. Clin. Exp. Microbiol.

[29] Wibowo M.H, Sahesty A, Mahardika B.K, Purwanto B, Lestariningsih C.L, Suardana I.B.K, Winaya I.B.O, Irine I, Suryanggono J, Jonas M, Murwijati T, Mahardika G.N (2019). Epizootiology, clinical signs and phylogenetic analysis of fowl adenovirus in chicken farms in Indonesia from 2018 to 2019. Avian Dis.

[30] Xu A, Pei Y, Zhang K, Xue J, Ruan S, Zhang G (2020). Phylogenetic analyses and pathogenicity of a variant infectious bursal disease virus strain isolated in China. Virus Res.

[31] Dey S, Pathak D, Ramamurthy N, Maity H.K, Chellappa M.M (2019). Infectious bursal disease virus in chickens:Prevalence, impact, and management strategies. Vet. Med. (Auckl).

[32] Mazengia H, Tilahun S.B, Negash T (2009). Incidence of infectious bursal disease in village chickens in two districts of Amhara Region, Northwest Ethiopia. Livest. Res. Ruler Dev.

[33] Khan R.W, Khan F.A, Farid K, Khan I, Tariq M (2009). Prevalence of infectious bursal disease in broiler in District Peshawar. ARPN J. Agric. Biol. Sci.

[34] Juranová R, Nga T, Kulíková L, Jurajda V (2001). Pathogenicity of Czech isolates of infectious bursal disease virus. Acta Vet. Brno.

[35] Latif I.K, Majed H.M, Sahar H (2014). Determine the weight of thymus, bursa of Fabricius and spleen and its ratio to body weight in some diseases of broilers. Mirror Res. Vet. Sci. Anim.

[36] Orakpoghenor O, Oladele S.B, Abdu P.A, Markus T.P, Andamin A.D, Esievo K.A.N (2021). Comparative pathological changes induced by very virulent infectious bursal disease virus infection in inoculated, sentinel pigeons and chickens. Open Vet. Sci..

[37] Islam M.T, Samad M.A (2004). Clinico-pathological studies on natural and experimental infectious bursal disease in broiler chickens. Bangladesh J. Vet. Med..

[38] Akter S, Bupasha Z.B, Alam M, Sarker M.S (2018). Infectious Bursal disease:A case compilation study in commercial broiler farms at Mirsarai, Chittagong, Bangladesh. Int. J. Adv. Res. Biol. Sci.

[39] Xu A.H, Sun L, Tu L.H, Teng Q.Y, Xue J, Zhang G.Z (2021). Experimental co-infection of variant infectious bursal disease virus and fowl adenovirus serotype 4 increases mortality and reduces immune response in chickens. Vet. Res.

[40] Rudd M.F, Heine H.G, Sapats S.I, Parede L, Ignjatovic J (2002). Characterisation of an Indonesian very virulent strain of infectious bursal disease virus. Arch. Virol.

[41] Hoque M.M, Omar A.R, Chong L.K, Hair-Bejo M, Aini I (2001). Pathogenicity of SspI-positive infectious bursal disease virus and molecular characterization of the VP2 hypervariable region. Avian Pathol.

[42] Islam M.N, Rashid S.M.H, Hoque M.F, Juli M.S.B, Khatun M (2008). Pathogenicity of IBDV related to outbreaks in the vaccinated flocks and the causes of vaccinated failure. J. Innov. Dev. Strategy.

[43] Kulsum U, Hossain M.N, Harun-Ur-Rashid S.M, Islam M.N, Salauddin M (2018). Pathological investigation of infectious bursal disease (IBD) in broiler at Dinajpur district. IOSR J. Agric. Vet. Sci.

[44] Ignjatovic J, Sapats S, Reece R, Gould A, Gould G, Selleck P, Lowther S, Boyle D, Westbury H (2004). Virus strains from a flock exhibiting unusually high mortality due to infectious bursal disease. Aust. Vet. J.

[45] Pandey M.K, Agrawal D.K, Mishra G.K, Gupta V, Raghuvanshi P.D.S (2021). Comparative haemato-biochemical and histopathological studies in birds inoculated with vaccine and field strain of infectious bursal disease virus. Indian J. Anim. Res.

[46] Mirzazadeh A, Asasi K, Mosleh N, Abbasnia M, Hachesoo A.B (2020). A primary occurrence of inclusion body hepatitis in absence of predisposing agents in commercial broilers in Iran:A case report. Iran. J. Vet. Res.

[47] Zahid B, Aslam A, Chaudhry Z.I, Akhtar R (2017). Biochemical and histopathological changes in immune and non-immune broilers after inoculation of field infectious bursal disease virus. Pak. J. Zool.

[48] Ogbe A.O, Audu Z, Kwaja E.Z (2020). Pathological diagnosis of infectious bursal disease in 24 weeks old vaccinated commercial laying hens in Kagarko, Nigeria:A case report. J. Dairy Vet. Anim. Res.

[49] Sharma J.M, Dohms J, Walser M, Snyder D.B (1993). Presence of lesions without virus replication in the thymus of chickens exposed to infectious bursal disease virus. Am. Assoc. Avian Pathol.

[50] Gao L, Li K, Qi X, Gao H, Gao Y, Qin L, Wang Y, Shen N, Kong X, Wang X (2014). Triplet amino acids located at positions 145/146/147 of the RNA polymerase of very virulent infectious bursal disease virus contribute to viral virulence. J. Gen. Virol.

[51] Nwagbo I.O, Shittu I, Nwosuh C.I, Ezeifeka G.O, Odibo F.J.C, Michel L.O, Jackwood D.J (2016). Molecular characterization of field infectious bursal disease virus isolates from Nigeria. Vet. World.

[52] Aliyu H.B, Hair-Bejo M, Omar A.R, Ideris A (2021). Genetic diversity of recent infectious bursal disease viruses isolated from vaccinated poultry flocks in Malaysia. Front. Vet. Sci.

[53] Deorao C.V, Rajasekhar R, Ravishankar C, Nandhakumar D, Sumod K, Palekkodan H, John K, Chaithra G (2021). Genetic variability in VP1 gene of infectious bursal disease virus from the field outbreaks of Kerala, India. Trop. Anim. Health Prod.

[54] Zhang W, Wang X, Gao Y, Qi X (2022). The Over-40-years-epidemic of infectious bursal disease virus in China. Viruses.

[55] Müller H, Mundt E, Eterradossi N, Islam M.R (2012). Current status of vaccines against infectious bursal disease. Avian Pathol.

